# Anomalous Cases

**Published:** 1863-10

**Authors:** Wm. H. Atkinson


### ANOMALOUS CASES.

BY WM. H. ATKINSON, M. D.

In general surgery and in medicine these have usually been either subjected to routine treatment without practical adaptation, or dismissed as hopeless and incurable.

The history of nearly every advance in surgery has proven that anomalous cases have fallen into the hands of young practitioners or the more adventurous experimenters, who thus instituted new methods and discovered unknown powers of recuperation or toleration in the living system, and hereby established the various improvements which make modern surgery so eminently conservative of life and limb.

Ligatures, amputations by clean cutting and sawing, exsection of bones, nerves, and diseased vessels, rhinoplasty, and enucleation have all had to pass the unwelcome baptism of opposition from nearly the whole corps of surgeons in the field at the time of the introduction of each. Therefore we should not regard it strange, nor evidence of a special perversity, if our humble attempts to enlarge the field of our usefulness in saving cases hitherto dismissed from the catalogue of curability and left to nature, or more recklessly and ruthlessly torn from their natural habitat for the lack of knowledge how to save and make them continue to subserve the purposes for which they were placed there. Even to this day, in some localities, the obsolete methods are still pursued in every variety of ignorance and misapprehension of the powers of the living system and principles of a surgery worthy of the name.

Probably in no department has stolid indifference and stubborn persistence in mutilations held more complete dominion than in that of what has been technically called "Operative Dentistry."

When any one has the temerity to step beyond that which is regarded as the do-able feats of the practice, he is fortunate indeed if he do not call down on his devoted head the maledictions of every "respectable" member of the profession, for daring to interpret nature in any other sense then the confined one, recognized by these self constituted and self-proclaimed "savans" of the established doctrine which should guide our honorable body.

There are many in the advanced ranks of the profession who owe much more to the inspirations that induced them to depart from the beaten track of authority, when this failed them in the treatment of anomalous cases, than to all the best instructions received from the so-called masters and authorities of the times.

I have been told in confidence of certain methods pursued with uniform success in out-of-the-way cases, which were kept from the light of the journals and confreres for no other reason than fear of ridicule by the reputedly wise among us. There needs no further testimony than this to prove that we as yet have no settled code of doctrine or principles upon which we may all safely depend in ambiguous forms of dental maladies.

All true authority resides in the perception of the fitness of things by the individual. So if any course of procedure uniformly results in desirable conditions, there can be no good reason why it should not be promptly ventilated for the better guidance of our whole co-fraternity in bringing about benign conditions in those for whom we have been employed.

It is in the memory of living men, who are yet in a vigorous practice, when it was regarded as unsafe and anomalous to attempt to fill approximal cavities in molar and bicuspid teeth with any encouragement that the work should prove a lasting benefit. And so I might say of each successive step in the wonderful progress of each branch of improvement in operative and mechanical dentistry. So that, if we were to take into the account and enumerate each operation that has at one time and another been deemed "anomalous" and "unheard of," we should be under the necessity of recounting every operation in the whole range of our specialty, except perhaps extraction and inserting teeth on rude ligatures or pivots.

With the great mass of those who acknowledge and call themselves dentists, all the more difficult and useful operations of permanent utility belonging to our profession would be regarded as anomalous and doubtful; while, on the other hand, we have a class among us to whom nothing is new or anomalous, or beyond their ability to demonstrate instanter, if we take their word for the demonstration. But I do not propose nor hope to enlighten all of the former class, nor to bring but few of the latter to the conviction of their puerility and entire lack of the very first elements of a true success in study and the practice of our noble calling.

I would say that some genius is requisite for the successful management of all cases which are anomalous to us, however commonplace and well defined and completely understood and managed soever they may be by others. The best off-hand definition of "genius" that comes to me now is "present use of your mental powers." The range of breadth, length, depth, and clearness will constitute the differences among *geniuses.*

The time is vividly within my recollection when the whole field of dental science was a void, and cases arising in it for practical treatment necessarily all anomalous to my perception and power to remedy. So my sympathy is natural, strong, and fraternal for all those in like conditions; and my chief purpose in penning this paper is to suggest the means, the use of which is to deprive the majority of operations in dentistry, and the surgery of the mouth of their anomalous character to my own apprehension, that others may be benefited by my labors, under the inspirations which the cases, and the circumstances under which they have fallen into my hands, have afforded me.

I. When an anomalous case is presented, inquire into its nature to the limit of present ability; and if there be no feasible method by which you may benefit it, cast in your mind the ability of others within the range of your acquaintance, and also the ability, in time and means, of your patient to avail himself of the skill you are about to commend to him; and if, after mature consideration of all the circumstances, you really think he can obtain a better service than you feel able to afford him, take measures to introduce him to the source of the supposed superior skill. But if for any reason he can not avail himself of your kindly advice, and you *feel* that you *can* improve his condition, set about it with a pains-taking and prayerful state of mind, having the *good* of the *patient* as the prime motive in every effort of anxious labor you may have to employ to effect your purpose.

II. Carefully note the circumstances of the case, and how your mind is exercised about it, with every part of the process you institute and the mutations of the whole case in your note-book for reference and further making up a judgment respecting the diagnosis where this is not at the time clear, or noting this and the prognosis for future confirmation or modification as time may prove, and you will be astonished how much more vividly the case will possess your perceptive powers and be retained by the memory, and how marvelously this record will become a focalizing point of facts which indicate the nature of the unfolding case.

III. Communicate the case and treatment to the first dentist you meet without omission or addition, and you will probably be surprised to find that even one whom you had regarded as far from "au fait" in such matters may obviously serve you, and hasten a happy result to the troublesome anomalous case.

IV. In all cases avoid the risk of complicating the case by injudicious and indecisive interference when you have reason to fear you can not signally benefit it.

General B. presented an anomalous case. It was a left superior second molar completely split antero-posteriorly, leaving the inner cusp and the palatal fang in one part, and the outer half of the crown and the buccal fangs in the other. The canals of all the fangs and the nerve chamber were excavated and filled by holding the two halves firmly together until the welding of the gold kept them in place. A hole was drilled through the largest portion of the tooth, countersunk at each end, into which a heavy golden bolt, headed to fit the countersink at the lingual surface, and a nut of like shape fitted on a thread at the other end at the buccal face of the tooth. The head was left long enough to seize with a pair of dressing forceps to maintain it firmly in position, while the nut was tightly driven down into the countersink for it, having been secured in the embrace of a pin vice with slide, making the tooth once more a firm unit.

The split was enlarged with broaches and excavators across the grinding face of the tooth and at the proximal margins, anterior and posterior, at which last two points it was beveled at the edges by means of a driving burr, somewhat larger than the broach used to enlarge the crack, down to the margin of the gum, into which the gold was securely welded simultaneously with the progress of filling the slot across the crown, after which, thin files, corundum tape burnishers, etc. assisted in completing a solid and secure filling which binds the tooth on every side, on which the patient ate without inconvenience from the first.

As before suggested, all cases in the beginning are anomalous to him who is forced to be his own instructor; and hence, the very best means of lessening the cases of necessarily anomalous character should be resorted to by all, that there may be less of sheer experiment and more of clear understanding of the basal laws, which indicate not only the possibles, but the probables of what may be safely attempted and successfully accomplished.

These means chiefly consist in a close scrutiny of the doctrines extant in books and in minds of the erudite in such matters, which, if consulted with the requisite freedom, fervency, zeal and frequency, the small among us will soon become great, novices be made wise, the clumsy dextrous, the vacillating and purposeless steady and determined, the contracted and selfish broad and generous, the contentious and misanthropic affable, courteous and fraternal, so that we shall soon see, acknowledge, and practice the necessity of sending to the highest sources of information all who are inquiring the way in which honorably to attain the very first rank in intelligence and capability that can be focalized in one mind. Make tutors and professors all of this class, and there will be no excuse for entering upon the responsibilities of an arduous practice without due preparation and fitness. And the difficulties in the way of success in the shape of anomalies will soon be annihilated by each following the special department that he is most capable to execute with a complete success, thus sweeping as with the broom of destruction all contentions other than the generous emulations that only intensify noble minds and prepare them for higher and more satisfactory rendering of daily duty to profession and people alike, elevating us far above the petty jealousies of low states of skill and association.

